# Corrigendum: Elucidation of the Signatures of Proteasome-Catalysed Peptide Splicing

**DOI:** 10.3389/fimmu.2021.755002

**Published:** 2021-09-23

**Authors:** Wayne Paes, German Leonov, Thomas Partridge, Annalisa Nicastri, Nicola Ternette, Persephone Borrow

**Affiliations:** ^1^ Nuffield Department of Clinical Medicine, University of Oxford, Oxford, United Kingdom; ^2^ York Cross-Disciplinary Centre for Systems Analysis, University of York, York, United Kingdom; ^3^ Nuffield Department of Clinical Medicine, The Jenner Institute, University of Oxford, Oxford, United Kingdom

**Keywords:** peptide splicing, proteasome, splicing mechanism, antigen processing, peptide epitopes

There were three minor errors in our original article.

1) The **Materials and Methods** section required additional detail about the search strategy implemented in Peaks and the discovery workflow to facilitate replication of the methodology. Two extra sentences have been added to expand on this, and a third sentence describing a step that was used in our previous HLA-I discovery workflow for spliced peptides, but that is not required in the interrogation of datasets derived from polypeptide digests, has been removed.

A correction has been made to **Materials and Methods, Discovery workflow for identification of non-spliced and spliced peptides**, paragraphs 1, 2 and 3:

Paragraph 1: “PEAKS *de novo* assisted sequencing was implemented for the assignment of non-spliced peptides derived from individual polypeptide sequences following proteasomal digest of each of the 25 precursor substrates, and no PTMs were included in the *de novo* search space”.

Paragraph 2: “Therefore, for DNUPs containing a total of ‘*n*’ leucine residues, all permutations (2^n^) of L/I variants were computed prior to *in silico* splicing – e.g. for *de novo* sequence LTSLTLKE originating from polypeptide precursor LTSLVRRATLKENEQIPK, 2^3^ combinations of the original *de novo* sequence would be computed (LTSLTLKE, LTSITLKE, ITSLTLKE, LTSLTIKE, LTSITIKE, ITSLTIKE, ITSITLKE, ITSITIKE) and each sequence input to the splicing algorithm”.

Paragraph 3: “The (*n*-1)^th^ fragment was first scanned for a contiguous match across the polypeptide, and when found, its corresponding splice partner fragment was scanned for a contiguous match within the remainder of the polypeptide sequence. Due to the lack of an applied false discovery rate (FDR) for identification of spliced peptides from *de novo* sequencing of LC-MS/MS spectra that were not matched to a pre-defined database, very short spliced peptide sequences (5-7 aa) were omitted from analysis, and only sequences with a length of 8 aa or greater were considered. *Trans*-spliced peptides were omitted from the analysis”.

2) The sequence of precursor polypeptide PP13 was erroneously given in **Table 1** as LQPQLIHLYYFDCFSESAIRNA, but this peptide in fact ended in ‘K’ and not ‘NA’. The PP13 sequence has now been amended to LQPQLIHLYYFDCFSESAIRK; and overall amino acid frequencies within polypeptide precursors in **Table 2** have been adjusted to reflect this minor change. The corrected **Table 1** and **Table 2** appear below.
3) In **Supplementary Table 2**, eight of the original undigested precursor polypeptide sequences were accidentally included in the non-spliced lists (PP7, PP8, PP16, PP17, PP19, PP20, PP21, PP25). These have now been removed. Four spliced peptides (EGCPMVVKF, ALIKPLPSV, FIRNLSFKCS, SFKCSEDDLKTVFAQFGAK) were also erroneously present in the non-spliced lists. Two of them have now been removed as they were 1-mer fusions (FIRNLSFKCS, SFKCSEDDLKTVFAQFGAK) which we did not consider in the manuscript, while the 2 cis-spliced peptides (EGCPMVVKF, ALIKPLPSV) have been added to the spliced peptide lists. The corrected **Supplementary Material** can be accessed via the link below.

**Table 1:** Synthetic polypeptides subjected to *in vitro* proteasomal digestion.

Polypeptide ID	Protein ID	Sequence	Length (aa)
PP1	sORF-encoded polypeptide: APITD1	SSCLPCPLSFEKFK	14
PP2	Q92879: CUGBP Elav-like family member 1	EGCSSPMVVKFADTQK	16
PP3	Q5CZC0: Fibrous sheath-interacting protein 2	LVSIQKSIVSRSPIMIDQ	18
PP4	P15882: N-chimerin	LTSLVRRATLKENEQIPK	18
PP5	sORF-encoded polypeptide: CD81	LPRFESRVCGHSLPSCTCP	19
PP6	HIV-1 CH529 enr: Vif	DQLIHLYYFDCFSESAIRK	19
PP7	P37275: Zinc finger E-box-binding homeobox 1	SLIPVNGRPRTGLKTSQCS	19
PP8	Q8TDU5: Putative vomeronasal receptor-like protein 4	HLPLIHILLLFTQAILVSS	19
PP9	HIV-1 IIIB: Vif	ALIKPKQIKPPLPSVRKLTE	20
PP10	HIV-1 CH390 enr: Vif	TADQLIHLYYFDCFSESAIRK	21
PP11	Q9NW13: RNA-binding protein 28	IRNLSFKCSEDDLKTVFAQFGA	22
PP12	HIV-1 CH945 enr: Vif	LADQLIHLYHFDCFTESAIRNA	22
PP13	HIV-1 CH945 enr: Vif mutant	LQPQLIHLYYFDCFSESAIRK	22
PP14	HIV-1 NL4-3: Gag	FGEETTTPSQKQEPIDKELYPLA	23
PP15	HIV-1 NL4-3: Gag	AAMQMLKETINEEAAEWDRLHPVHA	25
PP16	P46013: Antigen KI-67	KSWADVVKLGAKQTQTKVIKHGPQR	25
PP17	Q00887:Pregnancy-specific beta-1-glycoprotein 9	EMTDLYHYIISYIVDGKIIIYGPAY	25
PP18	HIV-1 IIIB: Cryptic ORF	VAAPRLLPCALQQAESCVERSPLALLS	27
PP19	P47989: Xanthine dehydrogenase/oxidase	PRKQLRFEGERVTWIQASTLKELLDLK	27
PP20	Q92608: Dedicator of cytokinesis protein 2	YLDTSSRGEQCEPILRTLKALEYVFKFI	28
PP21	Q5T7P8: Synaptotagmin-6	KLKDPSTLGFLEAAVKISHTSPDIPAEVQM	30
PP22	HIV-1 NL4-3: Pol	AELELAENREILKEPVHGAYYDPSKDLIAEL	31
PP23	Q14894: Thiomorphine-carboxylate dehydrogenase	ALTTKLVTFYEDRGITSVVPSHQATVLLFEPSNG	34
PP24	HIV-1 NL4-3: Pol	IRKVLFLDGIDKAQEEHEKYHSNWRAMASDFNLPPVVAKEIVAS	44
PP25	Q9H8V3: Protein ECT2	KALKTIKIMEVPVIKIKESCPGKSDEKLIKSVINMDIKVGFVKMESV	47

**Table 2:** Amino acid frequencies within synthetic polypeptides and combined HIV-1 and UniProt human proteomes.

Amino acid ^a^	Frequency in polypeptides (%)	Frequency in combined HIV-1/human proteomes (%)
A	7.16	8.25
C	2.65	1.37
D	4.56	5.45
E	7.49	6.75
F	4.23	3.86
G	3.09	7.07
H	2.76	2.27
I	7.65	5.96
K	8.31	5.84
L	11.24	9.66
M	1.63	2.42
N	1.63	4.06
P	6.19	4.7
Q	4.40	3.93
R	4.23	5.53
S	8.31	6.56
T	4.89	5.34
V	5.86	6.87
W	0.65	1.08
Y	3.09	2.92

a Amino acids are denoted using single letter code.

Values in the sentence of the results text stating the total number and percentages of non-spliced and spliced peptides have been amended accordingly. The corrected sentence in the **Results, The Relative Proportions of Unique Non-spliced and Cis-Spliced Peptides Generated by the Constitutive Proteasome Are Dependent on Precursor Peptide Length** is now as follows:

“Overall, we observed a total of 1,200 unique non-spliced (72.9%) and 446 *cis*-spliced (27.1%) peptides (**Figure 1A**)”.

**Figure 1:**
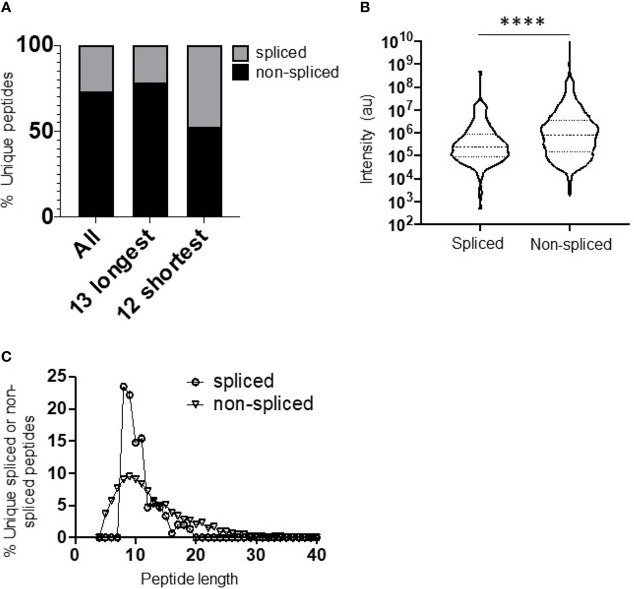
Diversity, abundance, and peptide length distribution of proteasome-derived spliced and non-spliced peptides. **(A)** Proportion of unique spliced and non-spliced peptides following a 2 h in vitro digestion of 25 self- and HIV-1-derived polypeptides (Table 1) by the constitutive proteasome. Proportions of spliced and non-spliced peptides within all unique peptides (n = 1,656), unique peptides originating from only the 13 longest polypeptide substrates (n = 1,337) and unique peptides originating from only the 12 shortest polypeptide precursors (n = 319) are shown. **(B)** Violin plots showing abundance of all unique spliced and non-spliced peptides as measured by LC–MS/MS intensity values. Median and quartile abundance values are indicated. A non-parametric unpaired Mann–Whitney t-test was used to determine whether abundance values differed between groups. ****P < 0.0001. **(C)** Length distributions of unique spliced (n = 135) and non-spliced (n = 900) peptides generated from within polypeptide substrates following a 2 h proteasomal digest.

The relevant panels of **Figure 1** and **Supplementary Figures 1, 2** have also been amended, and figure legends have been revised as needed. The corrected **Figure 1** and caption appear below.

“(A) Proportion of unique spliced and non-spliced peptides following a 2h *in vitro* digestion of 25 self- and HIV-1-derived polypeptides (**Table 1**) by the constitutive proteasome. Proportions of spliced and non-spliced peptides within all unique peptides (n=1,646), unique peptides originating from only the 13 longest polypeptide substrates (n=1,331) and unique peptides originating from only the 12 shortest polypeptide precursors (n=315) are shown”.

Given the large number of peptides observed overall, the changes made to the peptide lists in **Supplementary Tables 1, 2** have not had any impact on the conclusions drawn from the data, and the revised figure panels are not discernibly different from the originals. Importantly, none of these 12 peptides were used for any of the analyses in the manuscript from **Figure 2** onwards, as they all contained terminal amino acids of the polypeptide precursors.

In addition to these minor errors in the original version of our article, there was also an omission: the project accession numbers for the mass spectrometry datasets from the control undigested precursor substrates were not included in the Data Availability Statement. These have now been added.

The **Data Availability Statement** is now as follows:

“Mass spectrometry proteomics datasets have been deposited to the ProteomeXchange Consortium *via* the PRIDE partner repository (https://www.ebi.ac.uk/pride) with project accession numbers PXD025893 (for undigested precursor substrates) and PXD021339 (for proteasomal digests of precursor substrates). Computer scripts have likewise also been made privately available in GitHub and are available on request”.

The authors apologize for these errors and omissions, and state that they do not change the scientific conclusions of the article in any way. The original article has been updated.

## Publisher’s Note

All claims expressed in this article are solely those of the authors and do not necessarily represent those of their affiliated organizations, or those of the publisher, the editors and the reviewers. Any product that may be evaluated in this article, or claim that may be made by its manufacturer, is not guaranteed or endorsed by the publisher.

