# Supplementary material for: Corrigendum: Elucidation of the Signatures of Proteasome-Catalysed Peptide Splicing
Source: Front Immunol. 2021 Sep 23;12:755002. doi: 10.3389/fimmu.2021.755002 (PMC8496456; doi:10.3389/fimmu.2021.755002)
Supplement: Supplementary file 1 [file DataSheet_1.pdf]

Supplementary Figure 1

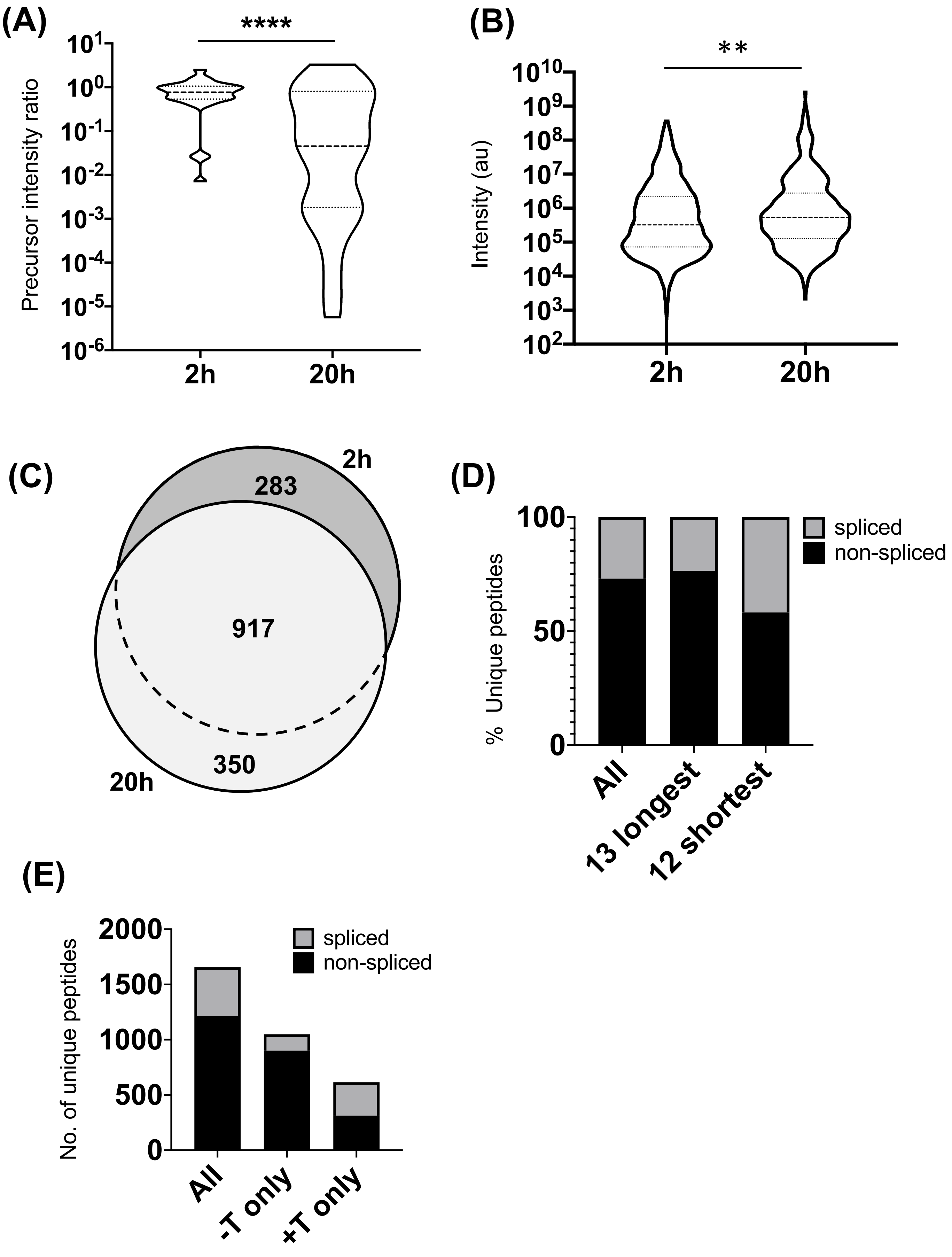

(F)

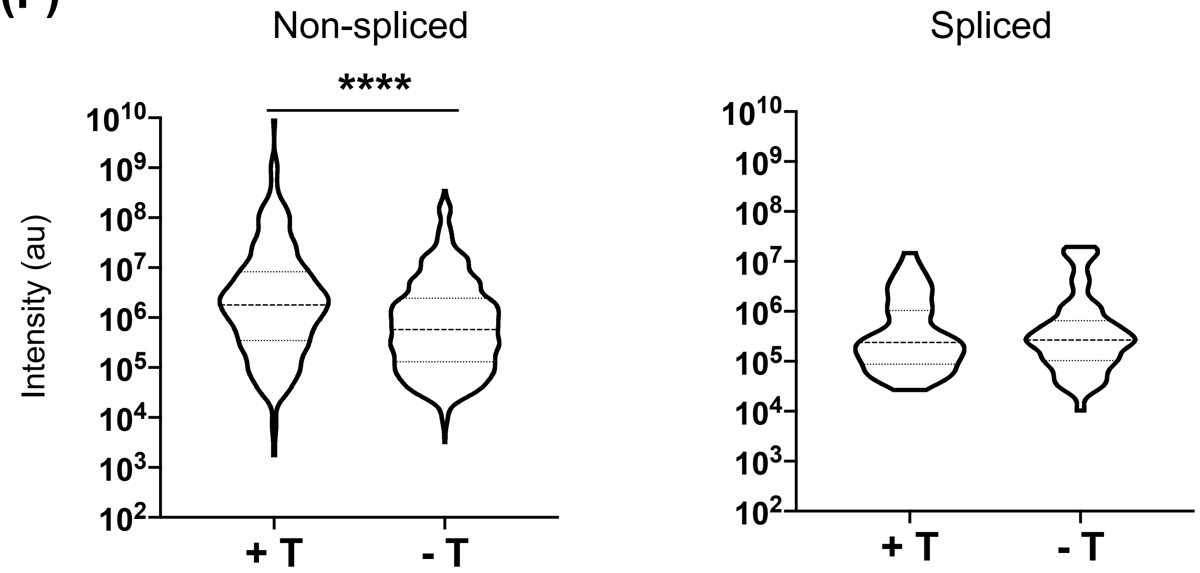

Supplementary Figure 1. Diversity and abundance of proteasome-derived spliced and non-spliced peptides

(A). Comparison of the abundance ratios (as quantified by LC-MS/MS intensity values) of full-length precursor polypeptides (**Table 1**) measured at the 2h and 20h timepoints relative to the undigested precursor at 2h. Median and quartile abundance ratio values are indicated. Statistical significance was calculated using a ratio paired t-test. **** P<0.001.

(B). Comparison of the relative abundance of total 5-8-mer peptides generated at 2h or 20h (as measured by LC-MS/MS intensity values). Median and quartile abundance values are shown. ** P<0.01.

(C). Area-proportional Venn diagram illustrating the diversity of constitutive proteasome-derived non-spliced peptides following *in vitro* digestion of precursor polypeptide substrates for 2h or 20h.

(D). Proportion of unique spliced and non-spliced peptides following 20h *in vitro* digestion of 25 self- and HIV-1-derived polypeptide sequences by the constitutive proteasome. Proportions of the unique peptides (n=1,739) generated from all 25 polypeptide substrates, unique peptides originating from only the 13 longest polypeptide substrates (n=1,414) and unique peptides originating from only the 12 shortest polypeptide precursors (n=325) are shown.

(E). Number of unique spliced and non-spliced peptide products identified following 2h *in vitro* digestion of 25 precursor polypeptides by the constitutive proteasome. Numbers of all unique spliced and non-spliced peptides, those originating from within the polypeptide substrate and not containing the terminal amino acid (-T) and those containing terminal amino acid(s) of the precursor substrate (+T) are shown.

(F). Violin plots showing abundance of the spliced and non-spliced peptide products containing terminal amino acids of precursor substrates (+T) and those originating from within the polypeptide substrate (-T), as measured by LC-MS/MS intensity values. Median and quartile abundance values are indicated. ****P≤0.0001. In (B) and (F), P<0.05 was used as the threshold for significance following a non-parametric unpaired Mann-Whitney t-test. .

Supplementary Figure 2

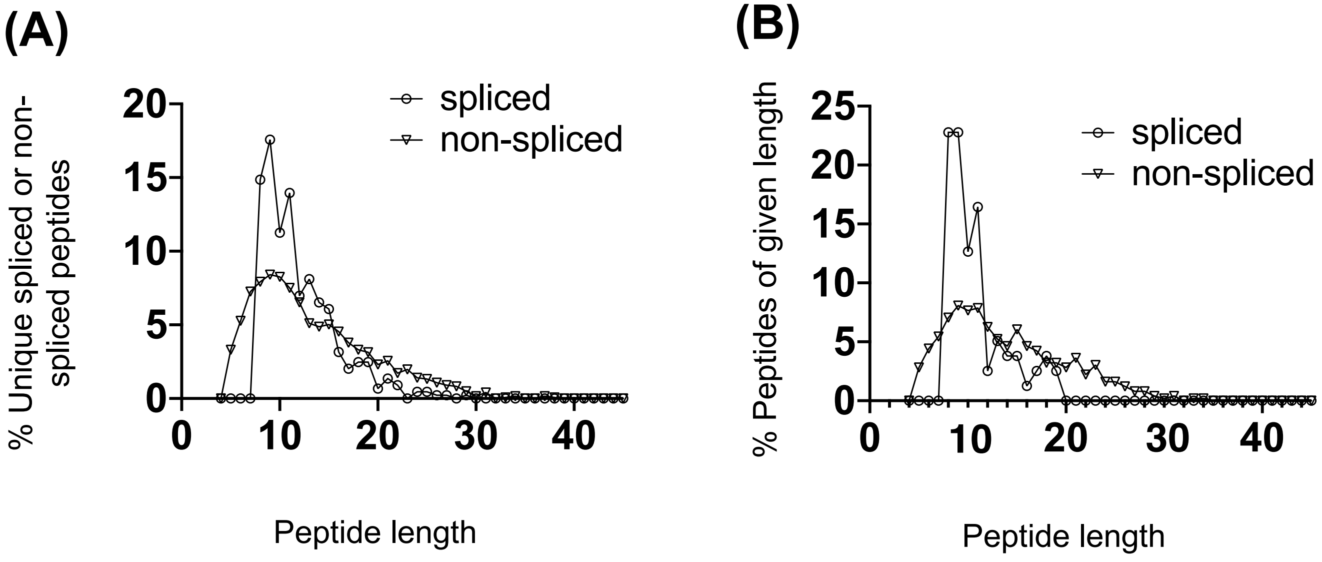

Supplementary Figure 2. Length distribution and abundance of peptide products generated from 2h *in vitro* proteasomal digests

(A). Length distributions of total unique spliced (n=446) and non-spliced (n=1,200) peptide products generated from all 25 precursor polypeptide substrates after a 2h *in vitro* digest.

(B). Length distributions of unique spliced (n=156) and non-spliced (n=608) peptides generated from the 5 longest precursor polypeptide substrates (30-47 aa in length) after a 2 h *in vitro* digest.
